# Effects of a Dietary L-Carnitine Supplementation on Performance, Energy Metabolism and Recovery from Calving in Dairy Cows

**DOI:** 10.3390/ani10020342

**Published:** 2020-02-21

**Authors:** Jennifer Meyer, Susanne Ursula Daniels, Sandra Grindler, Johanna Tröscher-Mußotter, Mohamadtaher Alaedin, Jana Frahm, Liane Hüther, Jeannette Kluess, Susanne Kersten, Dirk von Soosten, Ulrich Meyer, Erika Most, Klaus Eder, Helga Sauerwein, Jana Seifert, Korinna Huber, Jürgen Rehage, Sven Dänicke

**Affiliations:** 1 Institute of Animal Nutrition, Friedrich-Loeffler-Institut, Federal Research Institute for Animal Health, Bundesallee 37, 38116 Braunschweig, Germany; Jennifer.Meyer@fli.de (J.M.); Susanne.Daniels@fli.de (S.U.D.); Liane.Huether@fli.de (L.H.); Jeannette.Kluess@fli.de (J.K.); Susanne.Kersten@fli.de (S.K.); Dirk.von_Soosten@fli.de (D.v.S.); Ulrich.Meyer@fli.de (U.M.); Sven.Daenicke@fli.de (S.D.); 2 Institute of Animal Science, Functional Anatomy of Livestock, University of Hohenheim, Fruwirthstraße 35, 70593 Stuttgart, Germany; s.grindler@uni-hohenheim.de (S.G.); Korinna.Huber@uni-hohenheim.de (K.H.); 3 Institute of Animal Science, Feed-Gut Microbiota Interaction, University of Hohenheim, Emil-Wolff-Str. 8, 70593 Stuttgart, Germany; johanna.troescher@uni-hohenheim.de (J.T.-M.); seifert.jana@uni-hohenheim.de (J.S.); 4 Institute for Animal Science, Physiology and Hygiene, University of Bonn, Katzenburgweg 7–9, 53115 Bonn, Germany; taherala@uni-bonn.de (M.A.); sauerwein@uni-bonn.de (H.S.); 5 Institute of Animal Nutrition and Nutrition Physiology, Justus-Liebig-University Gießen, Heinrich-Buff-Ring 26-32, 35392 Gießen, Germany; Erika.Most@ernaehrung.uni-giessen.de (E.M.); Klaus.Eder@ernaehrung.uni-giessen.de (K.E.); 6 Clinic for Cattle, University of Veterinary Medicine Hannover, Foundation, Bischofsholer Damm 15, 30173 Hannover, Germany; Juergen.Rehage@tiho-hannover.de

**Keywords:** L-carnitine, dairy cow, performance, energy metabolism, lipomobilisation, clinical score, calving, parturition

## Abstract

**Simple Summary:**

Dairy cows develop metabolic diseases especially in the transition period due to high energy requirements for the process of calving, beginning milk production and, simultaneously, restricted feed intake capacity. L-carnitine is endogenously synthesised as an obligatory, quaternary amine for the initial step of ß-oxidation, but with the onset of lactation it is also excreted with milk, whereby its availability for other metabolic pathways might be limited. Supplemental L-carnitine might be able to fill in this apparent gap and to enhance the efficiency of ß-oxidation, whereby the magnitude of negative energy balance would be decreased. The present experiment mainly focused on the energy-consuming process of calving itself and on the energy metabolism during the first weeks of lactation.

**Abstract:**

Dairy cows are metabolically challenged during the transition period. Furthermore, the process of parturition represents an energy-consuming process. The degree of negative energy balance and recovery from calving also depends on the efficiency of mitochondrial energy generation. At this point, L-carnitine plays an important role for the transfer of fatty acids to the site of their mitochondrial utilisation. A control (*n* = 30) and an L-carnitine group (*n* = 29, 25 g rumen-protected L-carnitine per cow and day) were created and blood samples were taken from day 42 ante partum (*ap*) until day 110 post-partum (*pp*) to clarify the impact of L-carnitine supplementation on dairy cows, especially during the transition period and early puerperium. Blood and clinical parameters were recorded in high resolution from 0.5 h to 72 h *pp*. L-carnitine-supplemented cows had higher amounts of milk fat in early lactation and higher triacylglyceride concentrations in plasma *ap*, indicating increased efficiency of fat oxidation. However, neither recovery from calving nor energy balance and lipomobilisation were influenced by L-carnitine.

## 1. Introduction

Dairy cows are metabolically challenged during the transition period which is characterised by a negative energy balance due to high energy requirements for beginning milk production and an inadequate feed intake capacity [1]. Pancreatic insulin production decreases post-partum (*pp*) and induces a decrease in glucose utilisation by insulin-sensitive organs [2], whereby the mammary gland obtains additional glucose for milk production [3]. In response to the energy deficit, a mobilisation of body fat reserves occurs and thus, the concentration of non-esterified fatty acids (NEFA) increases in blood. When the capacity of the carboxylic acid cycle is exceeded, excess of acetyl-CoA resulting from NEFA degradation are utilised in ketogenesis, and ketone bodies, such as ß-hydroxybutyrate (BHB), are formed. Moreover, a fatty liver syndrome might evolve from an insufficient export of triacyglycerides (TG), which are synthesised from surplus NEFA [4]. An efficient utilisation of NEFA depends on an adequate L-carnitine availability for fatty acid transfer into the mitochondrial matrix as the site of their oxidation. Therefore, an insufficient L-carnitine availability at times of an increased energy requirement, such as shortly after calving, might exacerbate the pathophysiological events discussed above [5]. An insufficient L-carnitine supply was proposed as a limiting factor for fatty acid metabolism [1]. Recent studies with L-carnitine-supplemented dairy cows demonstrated, among other things, a decreased TG accumulation in liver [5,6] and increased BHB levels in plasma [5,7], which might hint at modulations in lipid and energy metabolism. 

The process of calving induces stress and pain [8], which further reduces feed intake on the day of parturition [9] and, therefore, increases energy deficit. Especially in this early period after calving, a sufficient L-carnitine availability might be important to compensate the loss of L-carnitine via milk [10] and facilitate recovery from calving. The extent of stress resulting from calving and the consequence on energy consumption might be evaluated by clinical variables such as respiratory rate, heart rate, primary rumen contractions and rectal temperature.

Therefore, the aim of the study was to investigate if L-carnitine-supplemented dairy cows were less affected by calving, and if L-carnitine supplementation affected *pp* lipid mobilisation with special attention to the first 72 h *pp*. 

## 2. Materials and Methods 

The experiment was carried out at the experimental station of the Institute of Animal Nutrition, Friedrich-Loeffler-Institut (FLI), in Braunschweig, Germany, in accordance with the German Animal Welfare Act approved by the LAVES (Lower Saxony Office for Consumer Protection and Food Safety, Germany) (AZ33.19-42502-04-16/2378).

### 2.1. Experimental Design

The experiment started individually for each cow with the expected day 42 ante partum *(ap)* and ended at day 110 *pp*. A total of 59 pluriparous German Holstein dairy cows, including eight rumen- and duodenum-cannulated cows, were assigned to two groups, a control (CON, *n* = 30) and an L-carnitine group (CAR, *n* = 29), balanced for numbers of lactation (2–5 lactations), body weight (568–1008 kg), body condition score (2.5–4.75) and fat-corrected milk yield of previous lactation. To circumvent ruminal degradation, the cows in CAR received 125 g of a rumen-protected L-carnitine product (Carneon 20 Rumin-Pro, Kaesler Nutrition GmbH, Cuxhaven, Germany) per cow and day, which was included in the concentrate feed. This amount corresponded to a daily L-carnitine intake of 25 g per cow and day. To balance the fat content of the L-carnitine product, CON obtained an equivalent fat product (BergaFat F-100 HP, Berg+Schmidt GmbH & Co. KG, Hamburg, Germany) as used for the rumen protection of the L-carnitine. The cows were kept in a free-stall barn with slatted floors and cubicles with rubber pads and were rehoused for calving in the calving pen, where a maximum of two cows were kept in one straw bedding box.

Both groups were fed with a partial mixed ration (PMR). Whereas the composition of roughage remained unchanged during the whole trial (70% maize silage and 30% grass silage), the proportion of roughage to concentrate was variable in accordance with the recommendation of nutrient and energy supply of the Society of Nutrition Physiology (GfE). Initially, up to day 1 *ap*, diets of 80% roughage and 20% concentrate were fed. The amount of concentrate was increased from 30% to 50% up to 14 days *pp* and from then on, 50% concentrate was constantly fed up to day 110 *pp*. The PMR was offered by feed-weigh troughs (Roughage Intake Control, System Insentec B.V., Marknesse, The Netherlands) and the supplementary, restricted, pelleted concentrate was provided via concentrate feeding stations (Insentec B.V., Marknesse, The Netherlands). Water was offered for ad libitum intake. The components and the chemical composition of roughages and concentrate feed are shown in Table 1.

### 2.2. Measurements and Sample Collection

Samples of the PMR were taken daily, whereas the concentrate feed was sampled once a week. The feed samples were pooled over four-week periods. Cows were equipped with ear transponders to enable the individual recording of feed and water intake. 

Before calving, the body weight (BW) was recorded once a week, and during the lactation period, twice a day with a scale situated between the milking parlour and barn. Once a week, the same person also evaluated the body condition score (BCS) according to Edmonson et al. [14] (5-point scale, 1 = lean, 5 = obese). The cows were milked at 5:30 am and 3:30 pm. During milking, the milk yield was recorded by a milk counter (Lemmer Fullwood GmbH, Lohmar, Germany). Milk samples were taken twice a week during morning and evening milking to analyse milk ingredients.

Blood samples were collected from *Vena jugularis externa* by needle puncture or for frequent sampling, *pp* by indwelling catheters (from 0.5 h until 12 h after calving). Prior to blood collection, 20 mL of blood were aspirated and discarded from a catheter which was flushed with 20 mL of 0.9% saline solution after the collection. Blood samples were taken at the following time points: day 42, 14, 7, 3 and 1 *ap*, 0.5, 1, 2, 3, 4, 6, 9, 12, 24, 48 and 72 h *pp*, day 7, 14, 21, 28, 42, 56, 100 and 110 *pp*. Furthermore, ultrasonic measurements according to Raschka et al. [15] were performed on day 42 *ap*, 72 h *pp*, day 42 *pp* and day 100 *pp* to estimate masses of adipose tissues (subcutaneous (SAT), retroperitoneal (RAT), omental (OAT), mesenteric adipose tissue (MAT)) by using a Mindray M5 Vet (Mindray, Shenzhen, China) diagnostic ultrasound system with a linear (6 MHz, Mindray 6LE5Vs) and a convex probe (3 MHz, Mindray 3C5s). 

### 2.3. Analyses

In accordance with the methods of the Association of German Agricultural Analytic and Research Institutes (VDLUFA) [16], the pooled feed samples were analysed for dry matter (method number 3.1), crude ash (method number 8.1), crude protein (method number 4.1.2), ether extract (method number 5.1.1), crude fibre (method number 6.1), neutral detergent fibre without ash (NDFom, method number 6.5.1) and acid detergent fibre without ash (ADFom, method number 6.5.2).

Milk ingredients (fat, protein, lactose and urea) were analysed by an infrared milk analyser (Milkoscan FT 6000^®^, Foss Electric, Hillerød, Denmark) and somatic cell count (SCC) was determined by a flow cytometric measurement (Fossomatic 500^®^, Foss Electric, Hillerød, Denmark).

For analyses of NEFA, BHB and TG, serum tubes were incubated for 30 min at 30 °C and the serum was separated via centrifugation of blood samples for 15 min at 15 °C and 3000 g (Varifuge 3.0, Heraeus, Hanau, Germany). The extracted serum was kept at a temperature of −80 °C until the day of measurement. Analyses were performed by a photometric method using the Eurolyser CCA 180 (Eurolyser Diagnostica GmbH, Salzburg, Austria). The insulin concentration in serum was determined by a sandwich-enzyme-linked immunosorbent assay (ELISA) (Bovine Insulin ELISA, 10-1201-01, Mecordia AB, Uppsala, Sweden) according to the manufacturer’s protocol. 

Furthermore, an automated blood gas and electrolyte analyser (GEM Premier 4000, Werfen, Kirchheim, Germany) was used for determination of sodium, potassium, chloride, hydrogen carbonate and calcium ions, glucose and lactate concentration, the temperature-corrected partial pressure of oxygen and carbon dioxide (TpO_2,_ TpCO_2_), oxygen saturation (sO_2_), total carbon dioxide, temperature-corrected pH, base excess (BE) and base excess in extra cellular fluid (Beecf) in heparinized blood which was collected in sample syringes (Werfen, Kirchheim, Germany). Total calcium and total phosphorous were measured spectrometrically in blood serum (Calcium AS III Arsenazo III colour test/Phosphorus (mono) ultraviolet (UV)-method kit, Greiner Diagnostic GmbH, Bahlingen am Kaiserstuhl, Germany). The concentrations of N^Ƹ^-trimethyllysine (TML), y-butyrobetaine (yBB), acetylcarnitine (ACA) and free carnitine (CA) were analysed in ethylenediamine tetraacetic acid (EDTA) plasma by a tandem mass spectrometry method according to Hirche et al. [17]. 

### 2.4. Clinical Examinations and Assessment of Health Status

Each cow was frequently clinically examined from 0.5 up to 72 h after calving according to the methods of Dirksen et al. [18]. To evaluate the collected clinical findings, a cumulative clinical score was created, in which the physiological level was defined as 0 and deviations with numbers increasing with severity (see Table A1, Appendix A). For example, a wet muzzle was regarded as physiological and received the score of 0, while a score of 1 was assigned to a dry muzzle. Clinical variables which might differ into both directions from the physiological range were treated differently. For example, a score of 0 was assigned to the physiological range of heart rate (65 to 85 beats per minute, bpm) while a deviation in direction of bradycardia or tachycardia was scored with 1, 2 or 3. Finally, the individual scores were cumulated and related to the maximum possible score of 29. Thus, a score of 29 represented a cow which deviated at maximum from the physiological value for each individual clinical parameter while a score of 0 indicated a clinically inconspicuous cow.

### 2.5. Calculations

The fat-corrected milk (FCM) was calculated according to Gaines et al. [19]:

FCM (4% fat) (kg/d) = (milk fat (%) × 0.15 + 0.4) × milk yield (kg/d)

Energy-corrected milk (ECM), milk energy, net energy requirement for maintenance (NEM) and net energy requirement for lactation (NEL) were calculated according to the Society of Nutrition Physiology (GfE) [11]:

$$\mathrm{ECM}\;(\mathrm{kg}/d)=\mathrm{milk}\;\mathrm{yield}\;(\mathrm{kg}/d)\times \frac{1.05+0.38*milk\;fat\;\left(\%\right)+0.21*milk\;protein\;\left(\%\right)}{3.28}$$

Milk energy (MJ NEL/kg) = 0.38 × milk fat (%) + 0.21 × milk protein (%) + 0.95

NEM (MJ NEL/d) = 0.293 × BW^0.75^ (kg)

NEL (MJ NEL/d) = (milk energy (MJ NEL/kg) + 0.086) × milk yield (kg/d)

Net energy balance (NEB) was calculated by using the following equation:

NEB (MJ NEL/d) = total NEL intake (MJ NEL/d) − (NEM (MJ NEL/d) + NEL (MJ NEL/d))

Gestational requirements were taken into account by addition of 13 MJ NEL/d for week six up to week 3 *ap* and of 18 MJ NEL/d for the last 3 weeks *ap*. 

According to Hurley et al. [20], the feed efficiency (FE) corresponds to the ratio of ECM and dry matter intake (DMI) (kg) and the residual energy intake (REI) was calculated by using the following formula:

REI (MJ NEL/d) = Total NEL intake (MJ NEL) − expected energy intake (EEI) (MJ NEL).

To calculate the EEI, a non-linear regression model was used, whereby significant variables and related coefficients were determined:

EEI (MJ NEL/d) = −95.98 + 6.09 × milk yield − 0.06 × milk yield^2^ + 17.61 × week of lactation − 1.96 × week of lactation^2^ + 0.07 × week of lactation^3^ + 0.08 × body weight.

The Revised Quantitative Insulin Sensitivity Check Index (RQUICKI) was calculated according to Caloni et al. [21]:

$$\frac{1}{\log Insulin\;\left[\frac{µU}{ml}\right]+logGlucose\left[\frac{mg}{dl}\right]+logNEFA\left[\frac{mmol}{l}\right]}$$

Anion gap was calculated by using following equation according to Brouwer et al. [22]:

(Na^+^) − ((HCO_3_^−^) + (Cl^−^))

The mass of SAT, RAT, OAT and MAT was calculated according to Raschka et al. [15] in consideration of different anatomic locations for ultra-sonographic measurements: twelfth rib at the level of the greater *trochanter* (R12), centre of *paralumbar fossa* from skin to *peritoneum* (AW3c), intertransverse space cranial to lumbar, intertransverse space where caudal pole of kidney is visible from skin to kidney margin away from transducer (KD3b), back fat thickness (BFT), centre of *paralumbar fossa* from skin to muscle margin away from transducer (AW3b), point of interception of a vertical line through the last *lumbar vertebra* and a horizontal line through the greater *trochanter* from skin to muscle margin away from transducer (AW1b), intertransverse space cranial to lumbar intertransverse space where caudal pole of kidney is visible from skin to *peritoneum* (KD2c).

SAT (kg) = −6.66 + 0.72 × R12 + 0.31 × AW3c,

RAT (kg) = −9.55 + 0.62 × R12 + 0.06 × KD3b,

OAT (kg) = −2.32 + 0.55 × BFT + 0.37 × AW3b and

MAT (kg) = −12.8 + 0.38 × AW1b + 1.73 × AW3b − 1.45 × AW3c + 0.07 × KD2c.

The sum of the masses of OAT, MAT and RAT results in the abdominal adipose tissue mass (AAT). Additionally, the changes in adipose tissue masses (FM (kg/d)) were calculated by the differences between the different time points, whereby negative values indicated a gain and positive values indicated a reduction of masses in the particular localisation. The corresponding release of energy (considering that 1 g fat results in 39.8 kJ gross energy [23], whereby 16% of them is lost as heat [24]) was calculated as follows:

$$\mathrm{Release}\;\mathrm{of}\;\mathrm{energy}\;[\mathrm{MJ}/d]=\frac{FM\;\left[\frac{kg}{d}\right]\times 39.8\left[\frac{kJ}{kg}\right]\times 0.84}{1000}.$$

### 2.6. Statistical Analyses

Before statistical analyses were carried out, weekly mean values were calculated for performance and feeding data. Analyses were performed using the MIXED procedure of SAS software (SAS Enterprise Guide 6.1, SAS Institute Inc., Cary, NC, USA) with the restricted maximum likelihood method (reml). The model included the group (G, CON or CAR), the time (T) and the interaction between G and T as fixed factors. For blood variables, the values of day 42 *ap* were considered as covariables. The choice of covariance structure (compound symmetry, autoregressive or unstructured) for the respective parameter were based on the smallest Akaike information criterion for a finite sample size (AICC). Effects were assumed as significant when *p*-values were equal or smaller than 0.05. Least Squares (LS) Mean comparisons were carried out with the Tukey–Kramer test.

The relationships between REI and NEB, NEB and energy release from adipose tissue were evaluated by linear regressions for each experimental group. Additionally, linear regressions for the relationship between REI and NEB for cows in negative NEB and positive NEB (independent of treatment groups) were carried out and slopes were compared. All results are given as LS Means and additionally, the standard deviation is listed in the text. In tables and figures, the pooled standard error (PSE) of LS Means over both groups and all time points is indicated.

## 3. Results

Initially, CON included 30 cows and CAR only 29 cows, because of one non-pregnant cow. In total, five cows left the experiment before day 110 *pp* due to health problems. In CON, one cow had to be excluded in the first week of lactation because of a displaced abomasum with perforation. During the second week of lactation, two cows from CAR left due to high-grade lameness and atypical recumbency, respectively. In CON, one cow developed *pneumonia*, fatty liver, *pyometra* and a proliferative *glomerulonephritis* struck due to an impairment of general health status in week 8 of lactation, and a second cow in this group had to be excluded in week 8 of lactation because of a teat injury. In total, 27 cows in CON and 27 cows in CAR finished the entire trial. Results, which are not presented in this section, are presented in Appendix A.

### 3.1. Feed Intake 

**Figure 1:**
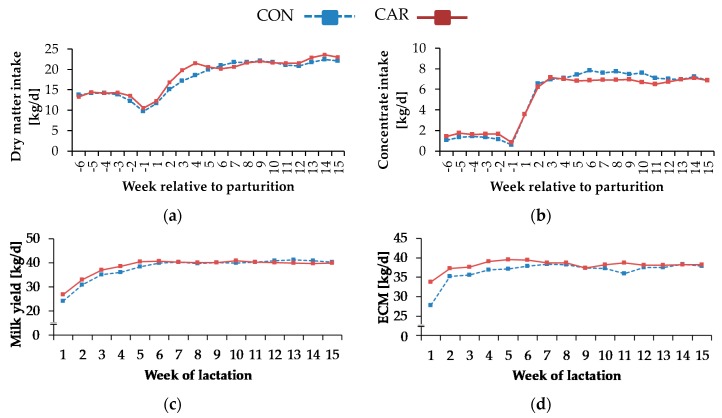

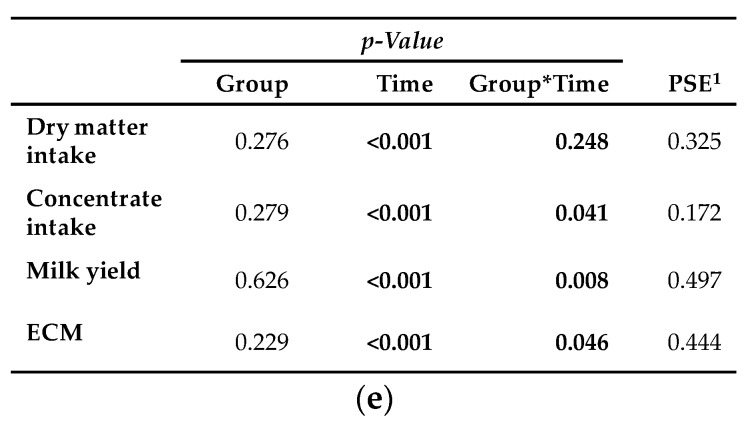
(**a**) Dry matter intake, (**b**) concentrate intake, (**c**) milk yield and (**d**) energy-corrected milk (ECM) of cows fed a non-supplemented (control, CON) or an L-carnitine-supplemented diet (25 g/d from 6 weeks *ap* up to week 15 *pp*, CAR). (**e**) Data statistics. Data are given as Least Square (LS)-Means. ^1^ pooled standard error.

Water intake decreased from week 6 *ap* until week 1 *ap* by 29% to the minimal level of 33 ± 2.64 kg/d (Table A2, Appendix A). Afterwards, it rose by 156% until week 5 *pp* to reach a level which remained unchanged until the end of trial (*p*_T_ < 0.001). Also, for DMI, a time-dependent variation was found (*p_T_* < 0.001) (Figure 1a). It dropped by 25% from week 6 *ap* until week 1 *ap*. Afterwards, there was a continuous increase by 100% until week 5 *pp*. This level remained unchanged until the end of the experiment. The concentrate intake (Figure 1b) was differently affected by L-carnitine supplementation over time (*p*_G*T_ = 0.041). From week 6 *ap* until week 2 *ap*, concentrate intake in CAR was on average 29% higher than in CON. Both groups reached the minimum level of 0.6 (CON)/0.8 (CAR) ± 0.12 kg/d in week 1 *ap*, which was not significantly different between both groups. In the following, there was a steep ascent until week 3 *pp* up to a 10 times higher level, which was kept until week 15 *pp*, whereby CON was 11% higher than CAR from week 5 until week 11 *pp.*

### 3.2. Milk, BW and BCS 

Progression of milk yield (*p*_G*T_ = 0.008), milk fat (*p*_G*T_ = 0.043) and protein (*p*_G*T_ < 0.001) was differently affected by L-carnitine supplementation over time. First, there was a slight increase in milk yield (Figure 1c). CON reached a first peak in week 6 *pp*, whereas this had occurred already in week 5 *pp* in CAR. Milk yield in CAR was on average 7% higher than in CON in the first 5 weeks *pp*. In the following, milk yields between both groups and between the time points were not significantly different.

**Figure 2:**
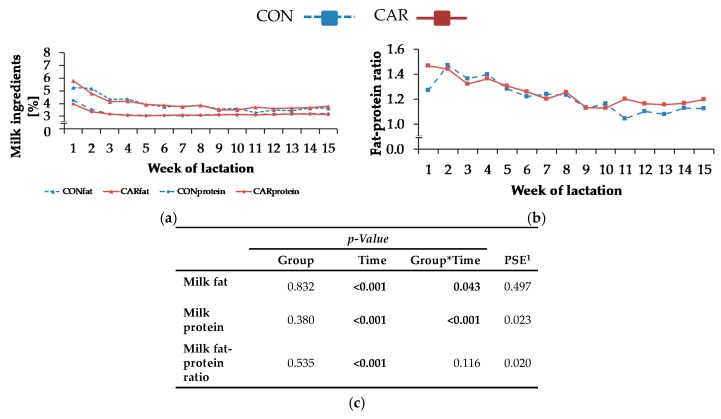
(**a**) Milk fat and protein and (**b**) fat-protein ratio in milk of cows fed a non-supplemented (CON) or an L-carnitine-supplemented diet (25 g/d from 6 weeks *ap* up to week 15 *pp*, CAR). (**c**) Data statistics. Data are given as LS-means. ^1^ pooled standard error.

Maximum milk fat content of 5.3% (CON)/5.8% (CAR) ± 0.19% was detected in week 1 of lactation (Figure 2a). In CON, it decreased by 18% from week 2 to week 3 of lactation, whereas milk fat in CAR began to fall from week 1 and decreased by 29% until week 3 of lactation to the same level as CON. Milk fat contents remained at this level for the rest of the experiment. Also, the highest level of milk protein (Figure 2a) occurred in week 1 of lactation. Initially, milk protein in CON reached 4.2% ± 0.05% and was therefore 7% higher than initial level of CAR (4.0% ± 0.05%). Until week 3 of lactation, there was a decrease by 25% in CON and 20% in CAR. Afterwards, both groups reached the same level and there were no differences until the end of the trial. For all other analysed milk ingredients, a typical time-dependent variation was found (*p_T_* < 0.001) which was, however, independent of treatment. The fat-protein ratio (Figure 2b) increased from week 1 to week 2 of lactation. Afterwards, it dropped with some fluctuations by 22% until week 9 of lactation. Thereafter, this level was kept constant until the end of the experiment. From week 1 until week 5 of lactation, milk lactose rose by 9% and stayed at this level for the rest of the trial (Table A3, Appendix A). Milk urea (Table A3, Appendix A) dropped from week 1 until week 3 of lactation by 41% and did not change further until the last week of the experiment. Until week 6 of lactation, there was a continuous decline of SCC and it remained unaltered for the rest of the experiment. The FCM (Table A3, Appendix A) was differently affected by L-carnitine supplementation over time (*p*_G*T_ < 0.05). There was an increase in FCM by 32% in CON and 11% in CAR from week 1 until week 2 of lactation, whereby FCM in CAR was on average 8% higher than in CON during the first 6 weeks of lactation. Thereafter, the treatment groups differed only slightly. The ECM (Figure 1d) changed in the same manner. Also, BW and BCS (Table A3, Appendix A) differed independently of the supplementation but were affected by the time relative to parturition (*p*_T_ < 0.001). 

The BW showed a continuous increase and reached a peak 2 weeks *ap*. There was a sharp decline from week 1 *ap* until week 1 *pp* by 17%. This level was then maintained until the end of the trial. The BCS followed a similar time course.

### 3.3. Energy Metabolism 

**Figure 3:**
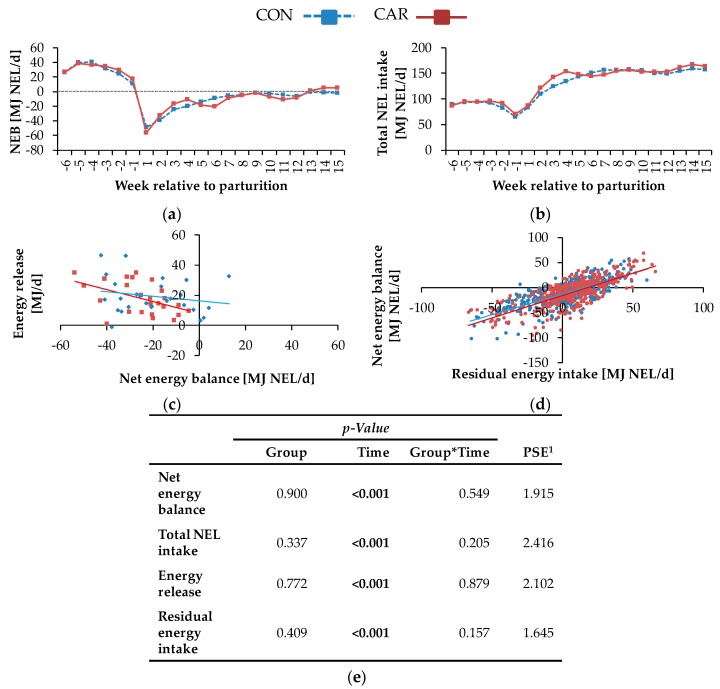
(**a**) Net energy balance (NEB), (**b**) total NEL intake of cows fed a non-supplemented (CON) or an L-carnitine-supplemented diet (25 g/d from 6 weeks *ap* up to week 15 *pp*, CAR), (**c**) regression of energy release from adipose tissue in dependence on net energy balance in the first six weeks of lactation. (*y_CON_* = −0.15 *^p^* ^> 0.05^*x* + 16.25, *r*^2^ = 0.041; *y_CAR_* = −0.40 *^p^* ^< 0.05^*x* + 8.00, *r*^2^ = 0.229; slopes were not significantly different (*p* > 0.05)), (**d**) and regression of net energy balance in dependence on residual energy intake from the first week until week 15 of lactation (*y_CON_* = 0.82 *^p^* ^< 0.05^*x* − 12.88, *r*^2^ = 0.549; *y_CAR_* = 0.89 ^*p* < 0.05^*x* − 15.67, *r*^2^ = 0.548); the slopes between CON and CAR were not significantly different (*p* > 0.05). (**e**) Data statistics. Data are given as LS-means in (a) and (b), ^1^ pooled standard error.

The NEL (Table A2, Appendix A) was differently affected by L-carnitine supplementation over time (p_G*T_ = 0.045). The initial level of NEL in CAR was 22% higher than in CON. It increased in CON by 33% and in CAR by 15% until week 4 *pp*. This level remained unchanged until the end of the trial. Total NEL intake (Figure 3b) decreased from week 6 *ap* until week 1 *ap* by 24% to the minimum level of 68 ± 2.4 MJ, which was followed by a continuous increase until week 14 *pp* to the maximum level of 163 ± 3.7 MJ. This level remained unchanged until the end of the experimental time (*p*_T_ < 0.001). Also, for NEB, a time-dependent variation was found (*p*_T_ < 0.001) (Figure 3a). The initial level of NEB rose by 48% to the maximum level of 39.1 ± 3.04 MJ NEL/d until 5 weeks *ap*. In the following, there was a continuous decline. NEB decreased further with a sharp decline from 1-week *ap* until week 1 *pp* to −52.4 ± 3.67 MJ NEL/d. At this point, NEB reached a negative value for the first time and at once, the lowest level. Afterwards, there was an irregular ascent and the cows started to enter a positive NEB from week 13 *pp* onwards. In week 13 *pp*, NEB was 97 times lower than in week 6 *ap*. As NEB increased, the energy released from mobilised adipose tissue decreased in a more or less pronounced linear fashion (Figure 3c). In CON, 0.15 MJ were mobilised from adipose tissue per 1 MJ NEB, while a steeper increase of 0.40 MJ per MJ NEB was estimated for cows in CAR. Statistically, the slopes were not significantly different (*p* > 0.05) between both groups. The FE (Table A2, Appendix A) was differently affected by the supplementation over time (*p*_G*T_ = 0.045). The initial level of FE was 5% higher than in CON. In CAR, FE decreased continuously by 32% until week 4 of lactation, whereby FE in CON decreased more slowly than in CAR and only by 15% until week 4 of lactation. From 2 weeks until 4 weeks of lactation, FE in CON was on average 17% higher than in CAR. In week 5 of lactation, both groups reached the same level, which remained unchanged up to the end of the experiment. The initial level of REI (Table A2, Appendix A) was −0.9 ± 2.48 MJ NEL/d, and a minimum level of −2.9 ± 2.94 MJ NEL/d was reached in week 6 *pp*. In the following, there were no significant deviations, but the highest value of 7.5 ± 2.77 MJ NEL/d occurred in week 14 *pp*. This level remained unchanged until the end of the trial (*p*_T_ < 0.001). 

The feeding group-dependent regressions over the whole range of REI and NEB did not suggest treatment differences (Figure 3d). To answer the question of whether mobilising cows displaying a negative NEB differ in efficiency compared to those with a positive NEB, cows were regrouped according to negative or positive NEB. These NEB-dependent regressions of REI (*x*) on NEB (*y*) did not reveal significantly different slopes (*y_negative_* = 0.56*x* − 21.85, *r*^2^ = 0.326; *y_positive_* = 0.51*x* + 5.03, *r*^2^ = 0.325). Therefore, all data were merged to one regression. An increase in REI by 1 MJ was associated with an increase of the NEB by 0.86 MJ, independently of the L-carnitine supplementation (*y* = 0.86*x* − 14.23, *r*^2^ = 0.545). 

### 3.4. Clinical Findings and Clinical Cumulative Score

**Figure 4:**
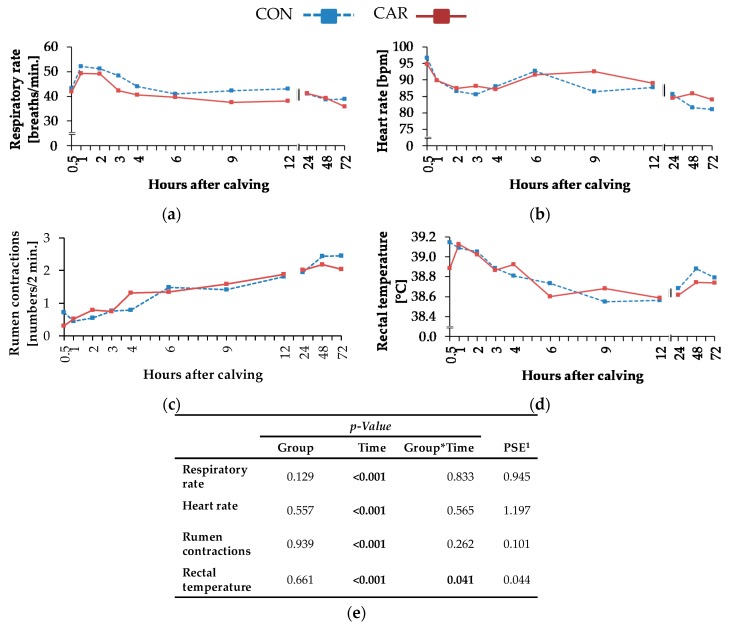
(**a**) Respiratory rate, (**b**) heart rate, (**c**) primary rumen contractions and (**d**) rectal temperature of cows fed a non-supplemented (CON) or an L-carnitine-supplemented diet (25 g/d from 6 weeks *ap* up to week 15 *pp*, CAR). Data are given as LS-means. (**e**) Data statistics. ^1^ pooled standard error.

**Figure 5:**
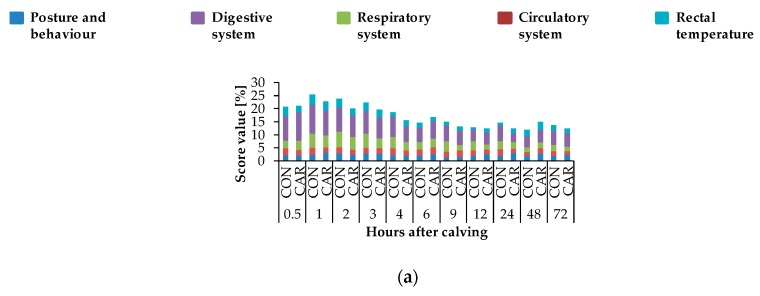

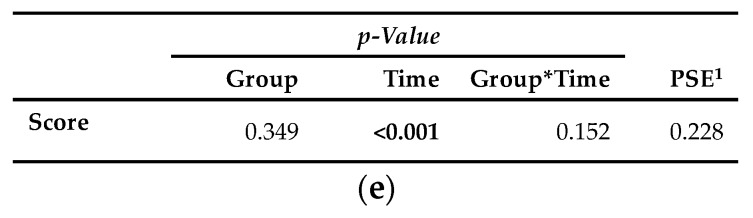
(**a**) Percentages of different categories (posture and behaviour, digestive system, respiratory system, circulatory system and rectal temperature) of cumulative score from maximum reachable score (29 points/100%) of cows fed a non-supplemented (CON) or an L-carnitine-supplemented diet (25 g/d from 6 weeks *ap* up to week 15 *pp*, CAR). (**b**) Data statistics. ^1^ pooled standard error.

Depending on the time after calving (*p*_T_ < 0.001), there was a steep increase of respiratory rate by 19% from 0.5 h until 1 h *pp*, where the maximum value of 50.7 ± 1.640 breaths/min was reached (Figure 4a). In the following, it decreased to a value which was 26% lower than the initial level until 72 h *pp*. Independently of treatments, the highest heart rate was detected at 0.5 h *pp* and decreased thereafter until 3 h *pp* (Figure 4b). Afterwards, heart rate increased within 6 h to a level which was only 4% lower than the initial rate. A continuous decrease followed until 72 h *pp* (*p*_T_ < 0.001). Primary rumen contractions (Figure 4c) reached the lowest level of 0.5 ± 0.15 numbers/2 min at 1 h *pp*. Until 72 h *pp*, there was a continuous enhancement to a level being 4 times higher than the initial level. The rectal temperature (Figure 4d) was differently affected by L-carnitine supplementation over time (*p*_G*T_ = 0.041). While rectal temperature in CON decreased continuously by 2% until 9 h *pp*, in CAR, it increased from 0.5 h until 1 h *pp* by 1% to a maximum level of 39.1 ± 0.10 °C, and decreased afterwards slightly until 12 h *pp* and finally increased again until 72 h *pp.* In CON, another decrease in the period from 9 h until 72 h *pp* was observed. Cumulative clinical score (Figure 5) reached a peak level after an increase by 16% from 0.5 h until 1 h *pp*. Subsequently, it dropped by 48% until 12 h *pp*. Afterwards, the score remained unchanged (*p*_T_ < 0.001). 

### 3.5. Blood Parameters

#### 3.5.1. Carnitine

**Figure 6:**
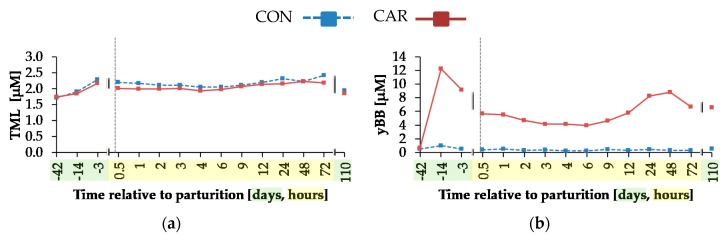

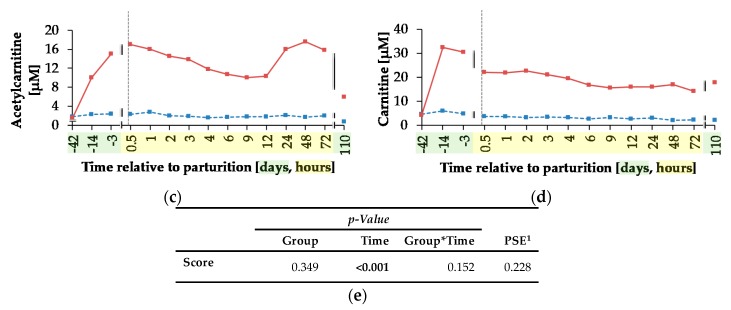
Concentrations of (**a**) N^ε^-trimethyllysine (TML), (**b**) y-butyrobetaine (yBB), (**c**) acetylcarnitine and (**d**) L-carnitine in plasma of cows fed a non-supplemented (CON) or an L-carnitine-supplemented diet (25 g/d from 6 weeks *ap* up to week 15 *pp*, CAR). (**e**) Data statistics. Data are given as LS-means. ^1^ pooled standard error.

Irrespective of treatment groups, the concentration of TML (Figure 6a) increased by 29% from day 42 *ap* until day 3 *ap*, decreased slightly until 0.5 h *pp*, and this level remained unchanged until 72 h *pp*. Afterwards, TML concentration dropped by 18% until 100 days *pp* (*p*_T_ < 0.001). The concentration of yBB (Figure 6b) was affected by L-carnitine supplementation over time (*p*_G*T_ < 0.001) and mean of yBB concentration was on average 15 times smaller in CON than in CAR, whereby the initial level of both groups was the same. In CON, there were no significant differences between the different time points. Until 14 d *ap*, there was a steep increase in CAR to a level which was 20 times higher than the initial level. Afterwards, there was a decrease until 0.5 h *pp* and this level persisted until 9 h *pp*. Until 48 h *pp*, the concentration of yBB increased by 90% and decreased in the following until 72 h *pp*. Dependent on treatments (*p*_G*T_ < 0.001), the mean of the ACA concentration (Figure 6c) was 6 times higher in CAR in comparison to CON, whereby for both groups, there was no significant difference between the initial level. The level of ACA concentration in CON did not alter during the whole trial. In CAR, the concentration of ACA increased to 12 times higher than the initial level until 0.5 h *pp*. In the following, it dropped until 12 h *pp* and then rose to the highest level of 17.63 ± 0.777 μM until 48 h *pp*. Afterwards, there was another decrease until day 110 *pp* to a level which was only 4 times higher than the initial level. Also, the CA concentration (Figure 6d) was differently affected by L-carnitine supplementation over time (*p*_G*T_ < 0.001). The initial level of the concentration of CA was the same in both groups, whereas the mean of CAR was on average 6 times higher than in CON. The concentration of CA level in CON was unchanged over the whole experiment. In CAR, CA concentration rose by 8 times until 14 d *ap* to decrease afterwards by 56% until 0.5 h *pp*. Thereafter, it dropped further by 36% until 72 h *pp* followed by a slight increase until day 110 *pp*.

#### 3.5.2. Serum Parameters with Relevance for Energy Metabolism

**Figure 7:**
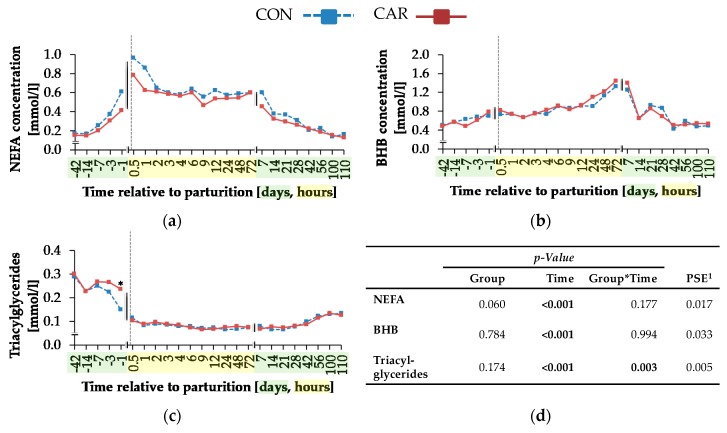
Serum concentrations of (**a**) non-esterified fatty acids (NEFA), (**b**) ß-hydroxybutyrate (BHB) and (**c**) triacylglycerides of cows fed a non-supplemented (CON) or an L-carnitine-supplemented diet (25 g/d from 6 weeks *ap* up to week 15 *pp*, CAR). (**d**) Data statistics. Data are given as LS-means. * symbol indicates significant differences between groups in a particular week according to the Tukey–Kramer test (*p* ≤ 0.05). ^1^ pooled standard error.

Regardless of treatment, the concentration of NEFA (Figure 7a) reached a peak of 0.87 ± 0.032 mmol/L at 0.5 h *pp*, which was more than 5 times higher than the initial value. Afterwards, a continuous decrease was noticed, and the initial level was reached again at day 110 *pp* (*p_T_* < 0.001). Also, irrespective of treatment, there was a steady increase of BHB concentration (Figure 7b) until 72 h *pp*, where the maximum level of 1.39 ± 0.071 mmol/L was reached. This was followed by a decrease from day 7 until day 14 *pp* down to the initial levels *(pT* < 0.001). The concentrations of TG (Figure 7c) were differently affected by the supplementation over time (pG*T = 0.003). CON and CAR started with comparable concentrations of TG contents in blood, which decreased until 0.5 h *pp* to a nadir. The concentrations of TG in CAR were on average 34% higher than in CON at days 3 and 1 *ap*, whereby these differences reached significance at day 1 *ap*. Subsequently, the low levels persisted up to the end of the trial.

The concentration of lactate (Table A4, Appendix A) remained unchanged at the first time points and increased from 1-day *ap* until 1 h *pp* to the maximum level of 1.09 ± 0.055 mmol/L, which was 2 times higher than the initial level. In the following, it dropped to the initial level again until day 14 *pp* and this level was kept constant until the end of the trial (*p*_T_ < 0.001). For glucose concentration (Table A4, Appendix A), a time-dependent variation was found (*p*_T_ < 0.001), its level remained unchanged during the last weeks of gestation and began to increase up to 3 days *ap*. At 2 h *pp*, glucose concentration reached the maximum level at 7.75 ± 0.136 mmol/L, which was over 2 times higher than the initial level. Afterwards, it dropped and reached the initial level again at 6 h *pp*. This level was kept until the end of the trial. No impact of the supplementation on insulin concentration (Table A4, Appendix A) was found. There was a steep increase of insulin concentration by 62% from the initial level until day 14 *ap* to a maximum value of 143.83 ± 9.339 pmol/L. Afterwards, there was a sharp decline. The minimum was reached 1 h *pp* and was 78% lower than the initial level of insulin concentration. At 4 h *pp*, a second peak occurred, at 72 h *pp*, there was another decrease and on day 110 *pp*, the initial level was reached again (*p*_T_ < 0.001). The RQUICKI (Table A4, Appendix A) varied only slightly over time (*p*_T_ < 0.001). There was a slow decline by 20% until 4 h *pp*, which was followed by a steady increase to maximum value at day 110 *pp*, which was 14% higher than the initial level. 

#### 3.5.3. Electrolytes

Data for electrolytes are presented in the Appendix A in Table A5. The initial blood sodium ion concentration increased by 2% from day 42 *ap* until day 1 *ap*, when the highest level occurred. Until 2 h *pp*, there was a decrease, whereby the initial level was reached again, and this level remained unchanged thereafter (*p*_T_ < 0.001). The Potassium ion concentration differed over time (*p*_T_ < 0.001), it increased from 6 h *pp* until 24 h *pp* by 6% to a maximum level of 3.98 ± 0.058 mmol/L. Afterwards, the concentration of potassium ions dropped until 7 h *pp*, where a minimal value was noticed and this level was kept until the end of the experiment. Independently of treatments, chloride ion concentration increased by 4% from the initial level until 0.5 h *pp*. This level persisted until 12 h *pp* followed by a sharp decline from 24 h *pp* until day 7 *pp* by 6% (*p*_T_ < 0.001). For hydrogen carbonate ion concentration in blood, a time-dependent variation was found (_pT_ < 0.001). It remained unaltered until day 14 *ap*. From there on, a decrease by 11% lasting up to 0.5 h *pp* was found. This level was kept until 4 h *pp*, when hydrogen carbonate ion concentration started to rise continuously by 17% until day 110 *pp*. The anion gap (*p*_T_ < 0.001) was unchanged at the first time points and increased by 32% from 48 h *pp* until 7 days *pp*. This level was kept constant and was followed by a decrease by 25% from day 2 *pp* until day 56 *pp*, where a minimum level of 8.32 ± 0.396 mmol/L was reached and persisted until the end of the trial.

#### 3.5.4. Calcium and Phosphorus

Data concerning concentrations of calcium and phosphorus are presented in the Appendix A in Table A6. Irrespective of treatment groups, calcium ion concentration was unchanged during the last weeks of pregnancy. From day 1 *ap* until 0.5 h *pp* there was a decrease by 15%, which was followed by a continuous increase to a maximum level of 1.29 ± 0.041 mmol/L at day 21 *pp*. This level was kept stable until the end of experiment (*p*_T_ < 0.001). Depending on time relative to parturition (*p*_T_ < 0.001), there was a steep increase by 8% for total calcium concentration from day 42 *ap* until day 14 *ap*. From day 7 *ap* until 1 h *pp*, it dropped by 19% and this level was kept constant until 2 h *pp*. From 3 h *pp* onwards until day 110 *pp*, the total calcium concentration increased to the maximum level. The mean of the ratio between calcium ions and total calcium concentration amounted to 0.59 irrespective of time and treatment. Total phosphorus concentration increased up to day 3 *ap* up to a maximum level of 1.79 ± 0.054 mmol/L. This was followed by a sharp decline by 45% to a minimum value at 0.5 h *pp*. This level was kept constant for a period of over 5 hours. Thereafter, a moderate increase from 6 h *pp* until day 110 *pp* was noticed (*p*_T_ < 0.001). The ratio of total calcium to total phosphorus concentration was influenced by the time relative to parturition (*p_T_* < 0.001) and remained unchanged until day 3 *ap.* From this time point until 0.5 h *pp*, an increase by 69% to a maximum was found. Afterwards, the ratio dropped continuously to the initial level at day 110 *pp*.

#### 3.5.5. Blood Gas Analyses

Data for blood gas analyses are presented in the Appendix A in Table A7 and for all of them, time-dependent variations were detected (*p*_T_ < 0.001). Independently of treatment groups, TpO_2_ and sO_2_ increased from the initial levels until 1 h *pp* by 46% and 23% respectively, and dropped afterwards to a level which was slightly smaller than the initial one until day 110 *pp*. The TpCO_2_ remained unchanged until shortly after parturition. From 72 h *pp* until day 14 *pp*, there was an increase by 9% to a maximum level that did not change until the end of the trial. The concentration of total carbon dioxide dropped by 9% from the initial level until 1 h *pp* to the minimum level of 27.77 ± 0.519 mmol/L. Afterwards, there was a continuous increase until day 110 *pp* to a peak level, which was 9% lower than the initial level. Temperature-corrected pH remained on the same level until 14 days *ap*. In the following, it dropped until 2 h *pp*, where a nadir occurred. Afterwards, there was an increase by 1% to the maximum level of 7.72 ± 0.005, which was reached on day 110 *pp.* BE and Beecf decreased from day 14 *ap* until 1 h *pp* by 79% and 69% respectively, and rose thereafter until day 110 *pp* to a level which was 8 and 4 times higher respectively, than the initial level.

### 3.6. Ultrasonography

**Figure 8:**
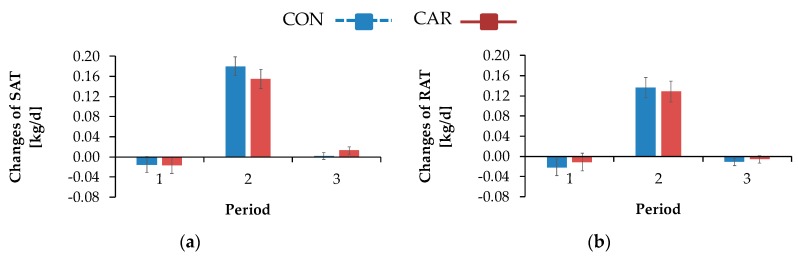

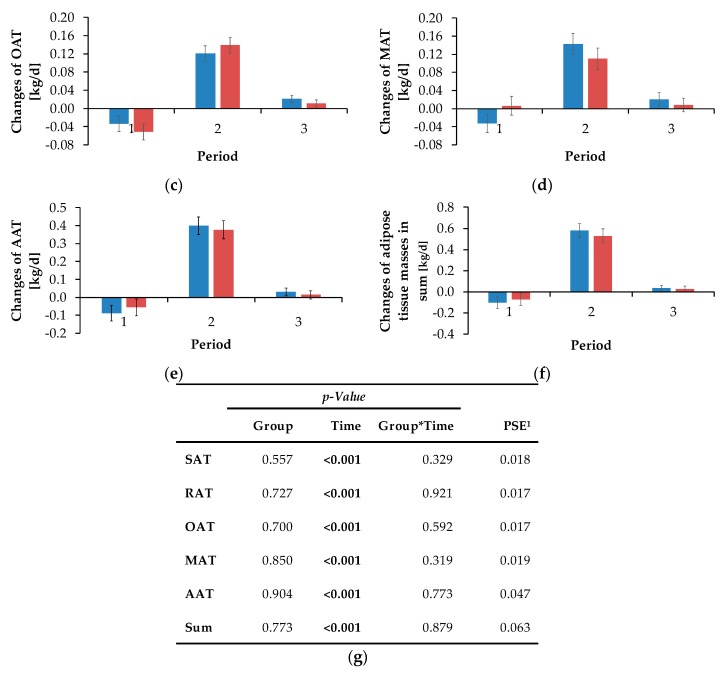
Changes in the (**a**) subcutaneous (SAT), (**b**) retroperitoneal (RAT), (**c**) omental (OAT), (**d**) mesenteric (MAT), (**e**) abdominal (AAT) adipose tissue masses and changes in masses of (f) SAT, RAT, OAT and MAT in sum of cows fed a non-supplemented (CON) or an L-carnitine-supplemented diet (25 g/d from 6 weeks *ap* up to week 15 *pp*, CAR), while positive values indicate a reduction and negative values indicate a gain of particular masses. (**g**) Data statistics. Period 1 = day 42 *ap* until day 3 *pp*, period 2 = day 3 *pp* until day 42 *pp*, period 3 = day 42 *pp* until day 100 *pp*. Data are given as LS-means ± standard error. ^1^ pooled standard error.

Irrespective of treatments, the adipose tissue masses (SAT, RAT, OAT, MAT, AAT) increased from day 42 *ap* up to 3 days *pp*, where all of them reached their maximum level (Table A8, Appendix A). After that, there was a decrease until day 42 *pp* and this level stayed constant until day 100 *pp*. The changes of adipose tissue masses in each localisation (SAT, RAT, OAT, MAT, AAT (Figure 8)) from day 42 *ap* until day 3 *pp* were in a slightly negative range. From day 3 *pp* until day 42 *pp*, the changes of adipose tissue masses reached positive and maximum values without exception. In the following (from day 42 *pp* until day 100 *pp*), the changes decreased but remained in a slightly positive range, whereby the changes of RAT reached a negative value again (*p*_T_ < 0.001). The daily energy release (Table A8, Appendix A) behaved in the same manner. 

## 4. Discussion 

The aim of the present study was to investigate the hypothesis that L-carnitine supplementation improves the energy status of the cow around calving with consequences on the energy-consuming puerperium, including the recovery from calving and the magnitude of negative energy balance. 

In doing so, clinical signs, milking performance, several indicators of energy metabolism including energy balance, adipose tissue dynamics, blood clinical-chemical traits and systemic carnitine levels, and electrolyte balance were evaluated with particular consideration of the first 72 h *pp*. 

As rations were designed on an isoenergetic basis and DMI remained unaffected by the L-carnitine supplementation, the resulting NEL intake followed a similar pattern. Carlson et al. [6] reported that DMI did not differ between non-supplemented cows and cows receiving 6 or 50 g of non-rumen-protected L-carnitine per day, whereas at a dosage of 100 g dietary L-carnitine per day, DMI was lower during the first two weeks of lactation. Based on these findings and on the present results, we conclude that a moderate L-carnitine supplementation neither stimulates nor depresses feed intake of transition cows. 

The REI represented the deviation of the EEI from total NEL intake, that could not be explained by the corresponding regression equation (Figure 3d). An individual positive REI represents a cow consuming more energy than expected and which is consequently less efficient compared to a cow showing a negative REI. As L-carnitine supplementation did not influence the REI, it might be concluded that energetic efficiency was not improved. Independent of dietary treatment, the NEB increased linearly and simultaneously with REI, suggesting the energetic efficiency to decrease with an increase in NEB. Here, cows in a negative NEB were as efficient as cows in a positive NEB, suggesting that efficiency during mobilisation is not different compared to periods of positive NEB. While the REI represented an estimate for the overall energetic efficiency, the contribution to the NEB by the energy mobilised from adipose tissues gave an indication for the energetic efficiency of lipomobilisation. 

The reason for the initially higher milk energy of L-carnitine-supplemented cows might be the simultaneously increased milk fat at this time point, which in turn did not result from differences in various time intervals from first milk sampling to the colostrum phase. Furthermore, L-carnitine had no effect on NEB. The absence of an improved NEB in CAR during early lactation might have been caused by the increase of energy output in milk in this group and the simultaneously unchanged NEL intake. Carlson et al. [6] found lower milk yields in the first 6 weeks of lactation for cows supplemented with 100 g L-carnitine/d, which might be due to the lower DMI as discussed above, whereas a supplementation with 6 or 50 g/d had no impact on milk yield. In the present experiment, milk fat and protein were also differently affected by L-carnitine supplementation over time. Carlson et al. [6] detected higher milk fat concentrations in pluriparous Holstein cows, which received a top dress mixture with 50 or 100 g L-carnitine/d from day 14 *ap* until day 21 *pp*, and also, Pirestani et al. [7] reported the same effect in L-carnitine-supplemented pluriparous Holstein cows (50 g/d/cow). Higher serum TG concentrations in CAR *ap*, which were utilisable for milk fat synthesis, might be one possible explanation for higher milk fat percentages in the first week of lactation in CAR. According to Carlson et al. [5], L-carnitine-supplemented cows might have a higher capacity to oxidise NEFA to BHB, which represent precursors for milk fat synthesis. 

While NEFA and BHB concentrations were not influenced by L-carnitine, the concentrations of TG were significantly higher in L-carnitine-supplemented cows shortly before calving. The ultrasonographic evaluation of the dynamics of different adipose tissues confirmed the view that L-carnitine did not influence energy metabolism and thus lipomobilisation. Also, FPR was not affected by L-carnitine supplementation. According to Buttchereit et al. [25], FPR is an adequate indicator for NEB during early lactation. Therefore, the unaltered FPR further supports the lack of effects by L-carnitine on lipomobilisation. Carlson et al. [5] detected a decreased TG accumulation in liver and increased serum BHB concentrations in abomasal L-carnitine-infused (20 g L-carnitine/d) and feed-restricted cows by simultaneously unaltered NEFA concentrations in comparison to abomasal water-infused and feed-restricted cows. This L-carnitine supplementation was nearly equal to the dietary supplementation in the present experiment, in which 25 g L-carnitine per cow and day were available for intestinal absorption in consequence of using a rumen-protected L-carnitine product (80% of dietary carnitine would be degraded in the rumen [6]). The higher levels of TG concentration in serum in CAR in the present study might be a hint at the mentioned decreased TG accumulation in liver. Also, serum NEFA concentrations were unaffected, but no increase of the BHB concentration in L-carnitine-supplemented cows was observed in the present study. This controversial result might be due to the difference between the physiological progression of a negative energy balance during the period of transition in the present experiment and the induction of this by an abrupt feed restriction outside this period, as in the trial of Carlson et al. [5]. These results indicated that in dietary L-carnitine-supplemented periparturient cows, an increase of ketogenesis was not associated with putative simultaneously decreased TG accumulation in liver. Furthermore, Pirestani et al. [7] detected a higher BHB concentration in cows receiving a combination of L-carnitine (50 g/d) and choline (60 g/d). Carlson et al. [6] also found a decreased TG accumulation in liver of periparturient cows, which received 6, 50 or 100 g of a non-rumen-protected dietary L-carnitine product per day. The BHB concentration in blood was higher in the 50 and 100 g groups, while NEFA concentrations were unaffected by L-carnitine supplementation. Apart from the fact that the extent of ruminal degradation of L-carnitine is unclear, the cows in the experiment of Carlson et al. [6] received anionic salts, minerals and vitamins from different sources, which might have had an effect, too. Furthermore, the L-carnitine supplementation in the experiment of Carlson et al. [6] started at 14 days *ap*, whereas the cows in the present study were already supplemented from day 42 *ap*. This longer adaptation period might have also been a reason for differences between the results of both studies. 

In the present experiment, the maximum NEFA concentration was reached at 0.5 h *pp*, when NEB was most negative and also fat mobilisation was at the maximum level. From day 100 *pp*, the initial NEFA concentration was reached again, which coincided with changing from a negative to a positive NEB. The increased BHB concentration went along with low glucose concentrations. The maximum BHB concentration occurred at 72 h *pp*. This might have been the time point, when the demand for glucose was higher than the capacity for gluconeogenesis in liver, resulting in an incomplete oxidation of NEFA—named ketogenesis [4]. Also, Carlson et al. [6] described that glucose in blood was unaffected by 6, 50 or 100 g of a dietary L-carnitine product. The concentration of glucose increased rapidly in the first hour *pp*, whereas insulin concentration reached the minimum level at this time point. Interestingly, the peak of glucose concentration in the present experiment occurred already at 2 h *pp* and at 12 h *pp*, the initial level was nearly reached again. According to Weber et al. [26], the concentration of glucose decreased rapidly after calving and also, insulin concentration decreased around calving, which corresponds to the results in the present experiment. The time-dependent changes in glucose, insulin and NEFA concentrations suggested a lower *pp* insulin sensitivity, as suggested by the lower RQUICKI that was, however, not influenced by L-carnitine supplementation. The significantly higher TG concentration in CAR on day 1 *ap* might be a hint at an increased hepatic export capacity for TG in the form of a very low density of lipoproteins in the blood. 

Interestingly, in the first week of lactation, milk protein was higher in CON than in CAR. La Count et al. [1] could not detect this effect in lactating Holstein cows receiving a ruminal or abomasal infusion of 6 g non-rumen-protected L-carnitine/d, whereas an abomasal infusion of a larger amount of L-carnitine (20 g/d) also resulted in lower milk protein concentrations in Holstein cows with provoked negative energy balance through feed restriction [5]. Erfle et al. [10] detected a negative correlation between milk carnitine and milk protein during the first weeks after calving in non-supplemented cows. A competition between milk protein synthesis and carnitine synthesis might exist, but is not very likely in the present case because precursors of carnitine synthesis derived from tissue protein catabolism [10] and from milk protein were lower in CAR. Supplementation with L-carnitine should minimise the need for L-carnitine precursors from protein catabolism and endogenous L-carnitine synthesis should also be lower. In the study of Carlson et al. [6], the percentage of milk protein tended to be higher in cows supplemented with 100 g L-carnitine/d than in cows supplemented with 50 g L-carnitine/d. In consideration of the present data, no conclusive explanation for this effect could be found. 

Most of the clinical signs and the cumulative clinical score were characterised by distinct dynamic profiles. Maximum heart rate of Holstein Friesian cows was detected directly during calving (Kovács et al. [27,28]). This effect resulted from exertions during parturition but also from loss of volume in the form of the calf and amniotic fluid. After this, peak heart rate remained on a high level from 0 h *pp* until 0.5 h *pp* and decreased until 1 h *pp*, which corresponded to the results of the present experiment. Georg et al. [29] found the heart frequency to increase 60–90 min *ap* in Holstein cows. The process of calving is stressful and goes along with pain for the dam [8]. Acute stress results in an activation of the sympathetic nervous system and concomitantly, a release of epinephrine from the adrenal glands causing an increase of heart rate [30]. Furthermore, the mentioned development of heart rate might be due to the decreased parasympathetic activity, which resulted in an increase of sympathetic activity [28]. Heart rate is closely related to metabolic oxygen consumption and consequently to energy expenditure [31]. Thus, an increased heart rate goes along with increased oxygen consumption and indicates enhanced energy expenditure. Based on the fact that both treatment groups showed similar patterns of heart rate kinetics during the first 72 h *pp*, the L-carnitine supplementation obviously failed to support energy metabolism in this particular period. 

Respiratory rate is closely involved in oxygen supply of the organism and consequently to heart rate, but also in the regulation of acid-base homeostasis. In the present study, respiratory rate increased to maximum level 1 h *pp* and was 26% higher at this time point than 72 h *pp*, which was also caused by exertions due to parturition. Srikandakumar et al. [32] detected an increase of respiratory rate and venous TpO_2_ in heat-stressed Holstein cows, whereby TpCO_2_ and oxygen saturation decreased. Taking into account that the calving process is a stress challenge, too, this corresponds to most of the results in the present experiment, where the maximum value of TpO_2_ occurred simultaneously with highest respiratory rate (1 h *pp*), whereby TpCO_2_ was on a relatively low level at this time point in comparison to the following time points (maximum level of TpCO_2_ in the first 72 h *pp* occurred when respiratory rate reached the highest level). Maximum sO_2_ also happened at 1 h *pp* in this experiment, in which the cows calved outside the summer months and therefore, more oxygen was in the air. SO_2_ also increased in Jerseys and Australian Milking Zebus, but not in Holstein cows under heat stress [32]. The minimum concentration of hydrogen carbonate ions, total carbon dioxide, BE and Beecf occurred simultaneously to the peak of respiratory rate, whereby temperature-corrected pH reached the minimum level 1 h later. An increased respiratory rate results in an increase of carbon dioxide elimination [32]. In the following, TpCO_2_ and total carbon dioxide concentration decrease, whereby the limit of bicarbonate buffer system in blood is exceeded [32]. Due to decreasing concentration of hydrogen carbonate ions, temperature-corrected pH in blood decreases, too [32]. The discussed regulatory mechanisms between respiratory rate and acid-base homeostasis were not modulated by L-carnitine, further supporting the view that the energetic situation shortly after calving remained unaffected by L-carnitine. 

Numbers of primary rumen contractions decreased due to a depressed DMI until week 1 *ap*. Also, Kandylis et al. [33] ascribed decreased ruminal motility to a decreased feed intake in sulfur-supplemented ruminants. Jørgensen et al. [34] detected decreased rumen motility in hypocalcaemic cows at a Ca^2+^ concentration of ≤1.0 mmol/L in blood. In the present experiment, Ca^2+^ concentration in blood was at 1.05 mmol/L when rumen contractions reached the minimal level (1 h *pp*). According to Hove et al. [35], decreased gastrointestinal motility went along with decreased calcium absorption because calcium absorption in the small intestine depends on a continuous flow of substrate. 

Although the rectal temperature was differently affected by carnitine supplementation over time, the overall variation was minimum (38.58–39.11 °C) and inconsistently related to the dietary treatment. According to Suthar et al. [36] and Burfeind et al. [37], rectal temperature is influenced by parity, time of day and month, which might have contributed to the observed variation. 

Just like most of the discussed clinical variables (heart rate, respiratory rate, primary rumen contractions and rectal temperature) showed maximum values shortly after calving, the resulting cumulative clinical score also peaked at 1 h *pp*, whereby deviations in the digestive system at each time point provided the largest part of the cumulative clinical score and reached the highest percentage during the first h *pp*. 

A time-dependent variation was detected for the concentration of TML as the precursor for endogenous carnitine synthesis. In mammals, TML originates primarily from protein degradation [38] and was found in different proteins like cytochrome c, calmodulin, actin, myosin and histones [39]. In the present experiment, increased TML concentrations were detected until day 3 *ap* independent of L-carnitine supplementation. The lack of treatment differences might be due to the equal total NEL intake, the NEB and therefore, an assumed equal protein catabolism between both groups. Davis et al. [40] described a significant increase of TML concentration in plasma of starved rats as compared to fed rats, which possibly resulted from an induced proteolysis. Dairy cows in the transition period also have an insufficient energy supply, triggering not only fat mobilisation but also protein degradation as already indicated by the TML concentration in blood. 

The concentrations of yBB, ACA and CA were affected by L-carnitine supplementation. All of them remained unchanged in CON over the whole trial, but in CAR, there was a sharp increase of yBB and CA concentration until day 14 *ap*, which represented the period of adaptation to the L-carnitine supplementation. yBB originated from the conversion of TML occurring primarily in kidney in rats [41]. Rebouche et al. [42] detected a decreased hepatic activity of yBB dioxygenase—converting yBB into CA—in carnitine-supplemented rats, which could be the reason for the higher yBB concentrations in CAR. The decreased yBB and CA concentrations from day 14 *ap* until 0.5 h *pp* in both groups could result from variations in carnitine metabolism during this period. Also, according to Schlegel et al. [43], mRNA abundances of genes involved in fatty acid uptake, fatty acid oxidation, ketogenesis and of enzymes of carnitine synthesis as well as carnitine uptake in liver increased from week 3 *ap* until week 1 *pp* in dairy Holstein cows and thus, liver cells received sufficient amounts of carnitine for the transport of excessive amounts of NEFA. CA is released primarily as ACA from liver cells into the blood stream [44], which explains the persistent increase of ACA concentration at 0.5 h *pp*, when concentrations of yBB and CA decreased in blood, but increased in liver cells [43]. 

## 5. Conclusions

The results demonstrated that a supplementation of 25 g rumen-protected L-carnitine/cow/d resulted in higher milk yields in early lactation and also higher milk fat percentages in the first week of lactation. On the given data basis, L-carnitine supplementation failed to improve the cows´ recovery from parturition and to reduce the extent of negative energy balance. Furthermore, equal levels of fat mobilisation (based on ultrasound measurements), NEFA and BHB concentrations indicated that the L-carnitine supplementation in the present experiment did not enhance the energy availability and energetic efficiency. Interestingly, the export of TG from liver into blood seemed to be increased in L-carnitine-supplemented dairy cows during the last 14 days *ap*, which could have consequences on *pp* hepatic lipid accumulation.

## Figures and Tables

**Table 1:** Components and chemical compositions of concentrates and roughages in experimental diets.

	Concentrate Feed			Roughage	
	PMR ^1^	CON ^2^	CAR ^3^	Maize Silage	Grass Silage
Components (g/kg)					
Soybean meal	50	115	115	-	-
Rapeseed meal	200	150	150	-	-
Wheat	190	290	290	-	-
Maize	248	-	-	-	-
Barley	190	-	-	-	-
Dried sugar beet pulp	50	274	267	-	-
Urea	30	40	40	-	-
Soybean oil	10	15	15	-	-
Calcium carbonate	12	34		-	-
Mineral feed premix ^4^	-	40	40	-	-
Mineral feed premix ^5^	20	-	-	-	-
carnEon 20 RUMIN-PRO ^6^	-	-	83	-	-
BergaFat F-100 HP ^7^	-	42	-	-	-
Chemical composition					
Dry matter (g/kg)	884	885	886	363	368
Nutrient (g/kg DM ^8^)					
Crude ash	62	102	93	40	105
Crude protein	271	303	313	77	150
Ether extract	47	81	79	30	38
Crude fibre	60	82	81	204	261
A ^9^ NDF_om_^10^	162	194	186	414	503
Acid detergent fibre_om_^10^	87	111	116	237	296
Starch	447	275	278	337	-
Sugar	48	76	74	-	-
Energy (MJ/kg DM) ^11^					
NE_L_^12^	7.8	8.0	7.9	6.5	6.1
ME ^13^	12.2	12.7	12.7	10.8	10.3

Values are given as means. ^1^ partial mixed ration, ^2^ control group, ^3^ L-carnitine group, ^4^ ingredients per kg mineral feed according to the manufacturer’s specifications: 13 g Ca, 60 g P, 120 g Na, 60 g Mg, 6 g Zn, 4 g Mn, 1.25 g Cu, 100 mg I, 50 mg Se, 35 mg Co, 800,000 IU vitamin A, 100,000 IU vitamin D_3_, 2500 mg vitamin E, ^5^ ingredients per kg mineral feed according to the manufacturer’s specifications: 140 g Ca, 70 g P, 120 g Na, 40 g Mg, 6 g Zn, 5.4 g Mn, 1 g Cu, 100 mg I, 40 mg Se, 25 mg Co, 815,000 IU vitamin A, 100,000 IU vitamin D_3_, 1500 mg vitamin E, ^6^ rumen-protected formula for L-carnitine supplementation, Kaesler Nutrition GmbH, 27472 Cuxhaven, Germany, 20% L-carnitine, 80% fat, ^7^ fractionated palm fatty acid, Berg + Schmidt GmbH & Co. KG, 20099 Hamburg, Germany, palmitic acid min 98%, ^8^ dry matter, ^9^ assayed with a heat-stable amylase, ^10^ expressed exclusive of residual ash, ^11^ calculation based on equations of References [11,12,13], ^12^ net energy lactation, ^13^ metabolisable energy.

## Appendix A

**Table A1:** Definition of the cumulative clinical score. The physiological level was defined as 0. Clinical findings were assigned to five different categories. The lines between parameters indicate that only the parameter above or below were considered for the cumulative score. A maximum of 29 points could be reached.

Category	Parameter	Score	Meaning
Posture and behaviour	Ear position	0, 1, 2	Horizontal, tense and backwards, low
	Behaviour		
	Calm	0, 1, 2	Calm and attentive, calm, apathetic
	Nervous	0, 1, 2	Calm and attentive, nervous, hyper nervous
Circulatory	Muzzle	0, 1	Wet, dry
	Conjunctive		
	Hypoaemic	0, 1, 2	Pale rose, pale, cyanotic
	Hyperaemic	0, 1, 2	Pale rose, rose red, red
	Injected episcleral vessels	0, 1, 2, 3	No, low, medium, severe injected
	Washed out episcleral vessels	0, 1	No, yes
	Heart rate		
	Bradycardia	0, 1, 2, 3	65–85, 44–64, 23–43, <43 (bpm)
	Tachycardia	0, 1, 2, 3	65–85, 86–106, 107–127, >127 (bpm)
Respiratory	Respiratory rate		
	Bradypnea	0, 1, 2, 3	24–36, 15–23, 6–14, <6 (breaths/min)
	Tachypnea	0, 1, 2, 3	24–36, 37–49, 50–62, >62 (breaths/min)
	Pathological breathing sound	0, 1, 2, 3	None, low, medium, severe laboured breathing
Digestive	Primary rumen contractions		
	Hypoperistaltic	0, 1, 2	2–3, 1, 0 (numbers/2 min)
	Hyperperistaltic	0, 1, 2	2–3, 4, >4 (numbers/2 min)
	Rumen sound		
	Reduced	0, 1	Loud, less loud-silence
	Increased	0, 1	Loud, intensive loud
	Abdominal wall tension	0, 1, 2, 3	No, low, medium, severe increased tension
Rectal temperature	Rectal temperature		
	Hypothermia	0, 1, 2, 3	38–39, 37–37.9, 36–36.9, <36 (°C)
	Hyperthermia	0, 1, 2, 3	38–39, 39.1–40, 40.1–41, 41.1–42 (°C)

**Table A2:** Effects of an L-carnitine supplementation on water intake, net energy requirement for lactation (NEL), feed efficiency and residual energy intake (REI).

Week ^1^		Water Intake (kg/d)	NEL ^2^ (MJ NEL/d)		Feed Efficiency ^3^ (kg/kg)		Residual Energy Intake ^4^ (MJ NEL/d)
			CON ^5^	CAR ^6^	CON ^5^	CAR ^6^	
−6		46					
−5		47					
−4		45					
−3		44					
−2		43					
−1		33					
1		64	90.6	110.4	2.6	2.7	−0.9
2		75	114.9	121.6	2.5	2.1	3.3
3		79	116.2	122.7	2.2	1.9	5.3
4		83	120.3	127.4	2.2	1.8	4.1
5		84	121.1	129.0	1.9	2.0	−1.8
6		83	123.4	128.6	1.8	2.0	−2.9
7		83	125.1	126.2	1.8	1.8	−0.2
8		81	124.8	126.4	1.8	1.8	4.9
9		81	121.8	122.0	1.8	1.7	3.7
10		82	121.7	124.8	1.8	1.8	2.6
11		83	117.4	126.4	1.8	1.9	−0.4
12		82	122.2	124.3	1.8	1.8	0.5
13		82	122.4	124.5	1.7	1.7	7.2
14		81	125.2	124.9	1.7	1.6	7.5
15		79	123.5	124.6	1.7	1.6	1.6
*p*-value ^7^							
	G	0.767	0.229		0.432		0.409
	T	<0.001	<0.001		<0.001		<0.001
	G*T	0.115	0.045		0.045		0.157
PSE ^8^		1.331	1.452		0.035		1.645

Data are given as LS-means. ^1^ week relative to parturition, ^2^ NEL = (milk energy (MJ NEL/kg) + 0.086) * milk yield (kg/d), ^3^ feed efficiency = energy-corrected milk (kg/d)/dry matter intake (kg/d), ^4^ REI = NEL intake (MJ NEL)—expected energy intake (MJ NEL), ^5^ control group (non-supplemented diet), ^6^ L-carnitine group (L-carnitine-supplemented diet (25 g/d from 6 weeks *ap* up to week 15 *pp*)), ^7^ *p*-value for group (G), time (T) and interaction of G and T (G*T), ^8^ pooled standard error.

**Table A3:** Effects of an L-carnitine supplementation on body condition and milk parameters.

Week ^1^		BW ^2^ (kg)	BCS ^3^	FPR ^4^	Lactose (%)	Urea (mg/kg)	SCC ^5^ (log_10_/mL)	FCM ^6^ (kg/d)	
								CON ^7^	CAR ^8^
−6		713	3.3						
−5		730	3.4						
−4		740	3.4						
−3		750	3.4						
−2		762	3.5						
−1		759	3.5						
1		658	3.2	1.37	4.45	173	2.08	27.4	34.5
2		638	3.2	1.45	4.70	136	1.77	36.2	38.3
3		631	3.0	1.34	4.81	102	1.61	36.4	38.3
4		630	3.0	1.38	4.81	99	1.55	37.9	40.0
5		632	3.1	1.29	4.86	94	1.47	37.8	40.3
6		632	3.0	1.24	4.86	92	1.44	38.3	40.0
7		634	3.0	1.22	4.86	90	1.43	38.9	39.0
8		633	3.0	1.24	4.87	91	1.45	38.7	39.4
9		634	3.0	1.13	4.88	93	1.37	37.3	37.4
10		638	3.0	1.15	4.88	91	1.42	37.4	38.3
11		640	3.1	1.12	4.87	97	1.41	35.6	39.1
12		641	3.0	1.13	4.89	97	1.43	37.3	38.2
13		644	3.1	1.12	4.87	91	1.41	37.3	38.2
14		645	3.1	1.15	4.87	100	1.49	38.2	38.4
15		645	3.1	1.16	4.89	98	1.44	37.6	38.5
*p*-value ^9^									
	G	0.935	0.790	0.535	0.385	0.266	0.638	0.182	
	T	<0.001	<0.001	<0.001	<0.001	<0.001	<0.001	<0.001	
	G*T	0.145	0.935	0.116	0.631	0.278	0.995	0.029	
PSE ^10^		5.062	0.028	0.020	0.014	3.351	0.037	0.496	

Data are given as LS-means. ^1^ week relative to parturition, ^2^ body weight, ^3^ body condition score, ^4^ fat-protein ratio, ^5^ somatic cell count, ^6^ fat-corrected milk = (milk fat (%) * 1.05 + 0.4) * milk yield (kg/d), ^7^ control group (non-supplemented diet), ^8^ L-carnitine group (L-carnitine-supplemented diet (25 g/d from 6 weeks *ap* up to week 15 *pp*)), ^9^ *p*-value for group (G), time (T) and interaction of G and T (G*T), ^10^ pooled standard error.

**Table A4:** Effects of L-carnitine supplementation on lactate and glucose in blood, insulin in plasma and Revised Quantitative Insulin Sensitivity Check Index (RQUICKI).

Day/Hours ^1^		Lactate (mmol/L)	Glucose (mmol/L)	Insulin (pmol/L)	RQUICKI ^2^
−42		0.73	3.72	88.60	0.48
−14		0.54	3.66	143.83	0.44
−7		0.61	3.62	-	-
−3		0.53	3.51	76.83	0.46
−1		0.55	4.04	-	-
0.5		1.06	5.88	27.18	0.44
1		1.09	7.31	19.47	0.47
2		0.99	7.75	38.07	0.47
3		0.97	6.94	84.82	0.43
4		0.93	5.60	100.79	0.38
6		0.94	4.04	70.34	0.42
9		0.76	4.00	66.65	0.43
12		0.69	3.86	70.22	0.42
24		0.72	3.54	73.66	0.43
48		0.62	3.35	59.32	0.44
72		0.61	3.20	47.20	0.47
7		0.50	2.97	52.54	0.49
14		0.42	3.31	76.72	0.46
21		0.43	3.20	68.34	0.49
28		0.41	3.25	-	-
42		0.45	3.48	-	-
56		0.44	3.49	-	-
100		0.49	3.52	-	-
110		0.52	3.59	101.19	0.50
*p*-value ^3^					
	G	0.594	0.463	0.263	0.827
	T	<0.001	<0.001	<0.001	<0.001
	G*T	0.783	0.751	0.761	0.892
PSE ^4^		0.026	0.102	4.254	0.006

Data are given as LS-means. - = no measurement for this parameter at particular time point. ^1^ day/hours relative to parturition, ^2^ RQUICKI = 1/(logInsulin (µU/mL) + logGlucose (mg/dl) + logNEFA (mmol/L), ^3^ *p*-value for group (G), time (T) and interaction of G and T (G*T), ^4^ pooled standard error.

**Table A5:** Effects of L-carnitine supplementation on electrolytes in blood and on anion gap.

Day/Hours ^1^		Sodium (mmol/L)	Potassium (mmol/L)	Chloride (mmol/L)	Hydrogencarbonate (mmol/L)	Anion Gap ^2^ (mmol/L)
−42		140.20	3.85	106.20	29.11	1.45
−14		141.28	3.83	106.50	29.76	1.57
−7		142.35	3.82	108.18	29.09	1.52
−3		142.97	3.75	108.81	28.87	1.79
−1		143.59	3.81	109.23	28.12	1.42
0.5		141.89	3.84	110.26	26.58	0.98
1		141.28	3.87	109.63	26.47	1.02
2		140.89	3.81	109.68	26.81	1.02
3		141.42	3.77	109.79	26.88	0.99
4		141.71	3.81	109.74	27.11	1.03
6		141.96	3.76	109.00	28.26	1.18
9		141.69	3.82	109.12	27.70	1.36
12		141.69	3.88	109.07	27.79	1.41
24		141.47	3.98	109.41	27.11	1.20
48		141.08	3.85	108.07	28.50	1.30
72		140.35	3.84	106.90	28.37	1.39
7		140.80	3.62	103.07	30.31	1.62
14		140.30	3.66	103.40	30.11	1.46
21		139.52	3.80	103.10	29.03	1.46
28		139.75	3.71	102.93	30.22	1.50
42		138.87	3.77	103.56	29.72	1.26
56		139.37	3.69	103.89	30.88	1.32
100		138.43	3.77	102.30	31.36	1.32
110		139.19	3.69	102.79	31.63	1.48
*p*-value ^3^						
	G	0.393	0.733	0.594	0.303	0.638
	T	<0.001	0.019	<0.001	<0.001	<0.001
	G*T	0.306	0.722	0.967	0.980	0.407
PSE ^4^		0.129	0.025	0.234	0.240	0.168

Data are given as LS-means. ^1^ day/hours relative to parturition, ^2^ anion gap = (Na^+^) − ((HCO_3_^−^) + (Cl^−^), ^3^ *p*-value for group (G), time (T) and interaction of G and T (G*T), ^4^ pooled standard error.

**Table A6:** Effects of L-carnitine supplementation on calcium ions (Ca^2+^), total calcium (Ca), ratio of Ca^2+^ and Ca and ratio of Ca and total phosphorus.

Day/Hours ^1^		Ca^2+^ (mmol/L)	Ca (mmol/L)	Ca^2+^:Ca	Phosphorus (mmol/L)	Ca:Phosphorus
−42		1.32	2.15	0.63	1.45	1.58
−14		1.31	2.32	0.56	1.57	1.50
−7		1.29	2.32	0.56	1.52	1.60
−3		1.29	2.25	0.58	1.79	1.33
−1		1.24	2.09	0.57	1.42	1.58
0.5		1.05	1.93	0.53	0.98	2.25
1		1.05	1.87	0.56	1.02	2.01
2		1.06	1.88	0.58	1.02	2.02
3		1.05	1.86	0.57	0.99	2.17
4		1.05	1.91	0.56	1.03	2.06
6		1.08	1.94	0.56	1.18	1.81
9		1.11	1.97	0.57	1.36	1.67
12		1.11	2.02	0.56	1.41	1.62
24		1.09	1.94	0.57	1.20	1.89
48		1.15	1.98	0.58	1.30	1.65
72		1.18	2.08	0.73	1.39	1.65
7		1.26	2.12	0.88	1.62	1.43
14		1.29	2.29	0.56	1.46	1.68
21		1.29	2.27	0.57	1.46	1.68
28		1.27	2.27	0.56	1.50	1.62
42		1.28	2.18	0.59	1.26	1.83
56		1.27	2.24	0.57	1.32	1.81
100		1.26	2.27	0.57	1.32	1.83
110		1.24	2.32	0.52	1.48	1.65
*p*-value ^2^						
	G	0.542	0.807	0.822	0.638	0.363
	T	<0.001	<0.001	0.530	<0.001	<0.001
	G*T	0.954	0.950	0.516	0.407	0.334
PSE ^3^		0.009	0.019	0.061	0.025	0.037

Data are given as LS-means. ^1^ day/hours relative to parturition, ^2^ *p*-value for group (G), time (T) and interaction of G and T (G*T), ^3^ pooled standard error.

**Table A7:** Effects of L-carnitine supplementation on temperature corrected oxygen and carbon dioxide partial pressure (TpO_2_, TpCO_2_), oxygen saturation (SO_2_), total carbon dioxide (CO_2_), pH in blood and base excess (BE), base excess in extra cellular fluid compartment (Beecf).

Day/Hours ^1^		TpO_2_ (mmHg)	SO_2_ (%)	TpCO_2_ (mmHg)	CO_2_ (mmol/L)	pH	BE (mmol/L)	Beecf (mmol/L)
−42		30.82	61.37	46.91	30.47	7.41	3.35	4.72
−14		34.86	59.91	47.54	31.13	7.41	4.00	5.50
−7		36.69	63.35	47.73	30.46	7.40	3.26	4.69
−3		39.98	68.30	47.69	30.24	7.39	3.16	4.42
−1		40.69	69.21	47.72	29.48	7.39	2.17	3.45
0.5		42.59	72.01	45.52	27.86	7.38	0.99	1.95
1		45.13	75.58	46.04	27.77	7.37	0.83	1.73
2		43.70	72.70	46.73	28.13	7.37	0.95	2.04
3		42.75	72.25	45.67	28.18	7.38	1.23	2.24
4		42.22	71.47	45.53	28.41	7.39	1.58	2.58
6		41.34	71.94	45.75	29.57	7.40	2.79	3.89
9		42.03	72.52	45.72	29.03	7.39	2.19	3.17
12		40.19	70.53	44.82	29.10	7.40	2.29	3.36
24		41.85	73.31	45.38	28.41	7.39	1.67	2.54
48		41.09	71.20	46.49	29.83	7.40	2.91	4.08
72		40.16	69.48	46.96	29.70	7.39	2.70	3.89
7		36.05	63.82	50.49	31.75	7.39	4.11	5.76
14		35.30	61.36	51.24	31.58	7.38	3.73	5.41
21		35.49	59.71	49.80	30.46	7.38	2.76	4.26
28		35.40	58.82	51.01	31.70	7.39	3.91	5.53
42		33.69	57.40	49.28	31.16	7.39	3.36	5.06
56		32.54	57.21	50.98	32.35	7.40	4.47	6.31
100		29.98	53.93	49.82	32.83	7.41	5.10	6.92
110		29.68	56.31	48.09	33.07	7.42	6.31	7.45
*p*-value ^2^								
	G	0.745	0.758	0.315	0.283	0.455	0.453	0.412
	T	<0.001	<0.001	<0.001	<0.001	<0.001	<0.001	<0.001
	G*T	0.513	0.562	0.776	0.982	0.298	0.703	0.965
PSE ^3^		0.379	0.746	0.341	0.249	0.002	0.224	0.259

Data are given as LS-means. ^1^ day/hours relative to parturition, ^2^ *p*-value for group (G), time (T) and interaction of G and T (G*T), ^3^ pooled standard error.

**Table A8:** Effects of L-carnitine supplementation on ultrasonically determined subcutaneous (SAT), retroperitoneal (RAT), omental (OAT), mesenteric adipose tissue (MAT) and calculated total abdominal adipose tissue (AAT) as well as daily derived energy release for each of the mentioned fat depots.

					***p*-Value ^3^**			
**Adipose Tissue Masses (kg)**	**d 42 *ap***	**d 3 *pp***	**d 42 *pp***	**d 100 *pp***	**PSE ^4^**	**G**	**T**	**G*T**
SAT	12.7	13.7	6.7	6.3	0.729	0.402	<0.001	0.566
RAT	8.7	9.7	4.1	4.5	0.581	0.547	<0.001	0.953
OAT	13.5	15.8	10.2	9.2	0.674	0.535	<0.001	0.506
MAT	15.7	16.5	11.3	10.5	0.628	0.448	<0.001	0.611
AAT ^1^	37.9	42.0	25.7	24.2	1.717	0.472	<0.001	0.950
Daily energy release ^2^ [MJ/d]	d 42 *ap* up to 3 *pp*	d 3 *pp* up to 42 *pp*	d 42 *pp* up to 100 *pp*		PSE ^4^	G	T	G*T
SAT	−0.53	5.60	0.24		0.600	0.557	<0.001	0.329
RAT	−0.55	4.43	−0.28		0.576	0.727	<0.001	0.921
OAT	−1.41	4.35	0.55		0.567	0.700	<0.001	0.592
MAT	−0.44	4.22	0.48		0.647	0.850	<0.001	0.319
AAT^1^	−2.40	12.96	0.75		1.565	0.904	<0.001	0.773

Data are given as LS-means. ^1^ AAT = RAT + OAT + MAT, ^2^ energy release = (changes in adipose tissue masses (kg/d) * 39.8 (kJ/kg) * 0.84)/1000, ^3^ *p*-value for group (G), time (T) and interaction of G and T (G*T), ^4^ pooled standard error.
